# Learning from Practice: How East Lancashire Hospitals’ Pharmacy Service Has Embraced Information Technology

**DOI:** 10.3390/pharmacy8040177

**Published:** 2020-09-25

**Authors:** Alistair Gray, Clare Mackie, William Price, Emma Coupe, Susan Holgate, Emma Watson, John Eatough, Neil Fletcher, Karen Hodson, Efi Mantzourani

**Affiliations:** 1 Pharmacy Department, Royal Blackburn Teaching Hospital, Blackburn, Lancashire BB2 3HH, UK; clare.mackie@elht.nhs.uk (C.M.); william.price@elht.nhs.uk (W.P.); emma.coupe@elht.nhs.uk (E.C.); susan.holgate@elht.nhs.uk (S.H.); emma.watson@elht.nhs.uk (E.W.); john.eatough@elht.nhs.uk (J.E.); neil.fletcher@elht.nhs.uk (N.F.); 2 School of Pharmacy and Pharmaceutical Sciences, Cardiff University, Cardiff, Wales CF10 3NB, UK; HodsonKL@cardiff.ac.uk (K.H.); MantzouraniE1@cardiff.ac.uk (E.M.); 3 Primary Care, NHS Wales Informatics Service, Cardiff, Wales CF11 9AD, UK

**Keywords:** transfer of care, dedicated ward pharmacy, refer-to-pharmacy, hospital discharge, technology-enabled pharmacy services, medicines reconciliation, best possible medication history

## Abstract

The ethos of the pharmacy service at East Lancashire Hospitals NHS Trust (ELHT) could be described as ‘let’s make things better’. We have a history of innovation involving technology and people; one without the other does not work but together they are synergistic. The Trust currently does not have an electronic patient record (ePR) or electronic prescribing and medicines administration (ePMA), although we do have electronic prescribing for chemotherapy. However, like all Trusts, we have many electronic systems which offer interoperability, or can support making it easier for the pharmacy team to do a good job. This article describes the many fronts we have worked on over the last ten plus years. Taken individually, the elements cannot be considered as revolutionary; together, they have helped us develop and deliver the safe, personal and effective pharmacy service that we call dedicated ward pharmacy.

## 1. Defining the Problem

Transfer of care between care settings, including being discharged from hospital to the community, has traditionally been associated with a high risk of errors, exacerbated by miscommunication and unintended changes to medicines. Up to 70% of patients have either an error or an unintentional change to their medicines when their care is transferred [1]. Internationally, numerous interventions have been designed and delivered to try and reduce the risk of medication-related harm during hospital discharge.

**Figure 1:**
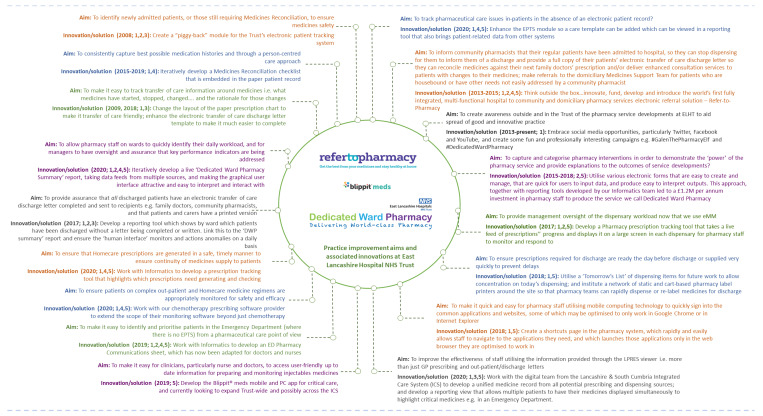
An overview of practice improvement aims/problems and associated innovations/solutions at East Lancashire Hospitals NHS Trust. Innovations include dates and are classified into categories: 1. improving communication; 2. generating data for performance evaluation/decision making; 3. addressing regulatory needs; 4. external patient driven; 5. addressing internal productivity and decision making.

This report presents the challenges we faced as a Trust in relation to transfer of care; how we applied incremental and radical innovations; and how they cumulatively improved our organisation’s capability to address evolving changes in the digital landscape we operate in today (Figure 1). We present these innovations mapped against the following classifications: 1. improving communication; 2. generating data for performance evaluation/decision making; 3. addressing regulatory needs; 4. external patient driven; 5. addressing internal productivity and decision making. We explain how we embraced the principles of managing innovation, driven by the ability to see connections, to spot opportunities and to take advantage of them [2]. Our innovations were locally developed in challenging healthcare environments and involved not only traditional indicators but many of the individuals that work for the Trust [3]. We conclude with a discussion of how our approaches had a broader impact on practice in healthcare.

## 2. Transfer of Care and Medicines Reconciliation (1. Improving Communication; 2. Generating Data for Performance Evaluation/Decision Making; 3. Addressing Regulatory Needs; 4. External Patient Driven)

The National Institute for Health and Care Excellence (NICE) defines medicines reconciliation as “the process of identifying an accurate list of a person’s current medicines and comparing it with the current list in use” and advises that “Medicines-related patient safety incidents are more likely when medicines reconciliation happens more than 24 h after a person is admitted to an acute setting” [4,5]. Medicines reconciliation is actually an international issue, with the World Health Organisation providing additional guidance, which NICE has built upon, that promulgates the concept of capturing a ‘best possible medication history’ (BPMH) [6]. 

**Figure 2:**
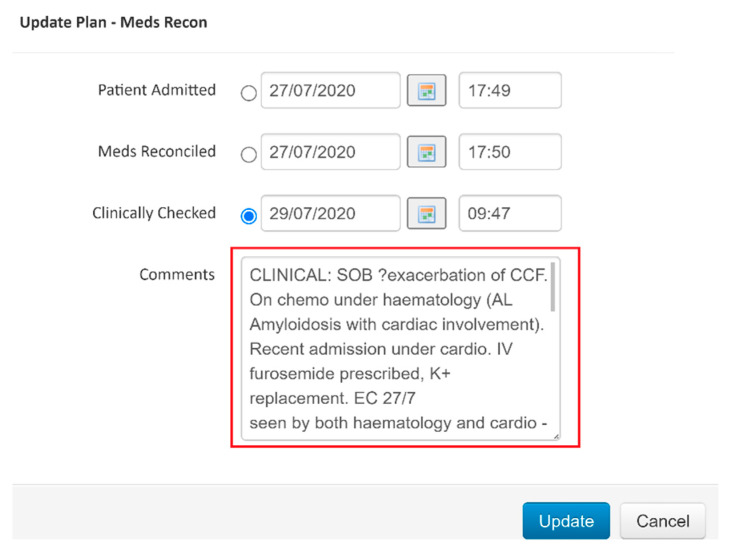
Medicines reconciliation flagging tool on the electronic patient tracking system.

Back in 2008, one of our main issues was how to efficiently identify patients who required medicines reconciliation, so that patients requiring the service could be quickly identified and without any duplication of effort. This was in response to the NICE patient safety guidance requiring that adults admitted to hospital should have their drug histories confirmed, and subsequently reconciled by pharmacy staff [7]. Serendipitously, the Trust had just introduced an electronic patient tracking system (EPTS) to identify which beds were occupied by whom and predict when wards were going to see patient transfers and discharges. We decided to take advantage of this initiative and it was arranged that a medicines reconciliation flag would be applied to each patient automatically on admission, and that the pharmacy team could change those flags’ statuses for individual patients to show whether the drug history had been confirmed, and also whether full reconciliation had occurred (Figure 2), with prescription charts matching reality, intentional changes documented, and any queries or anomalies resolved. The system allowed us to track and audit live performance of the team.

**Figure 3:**
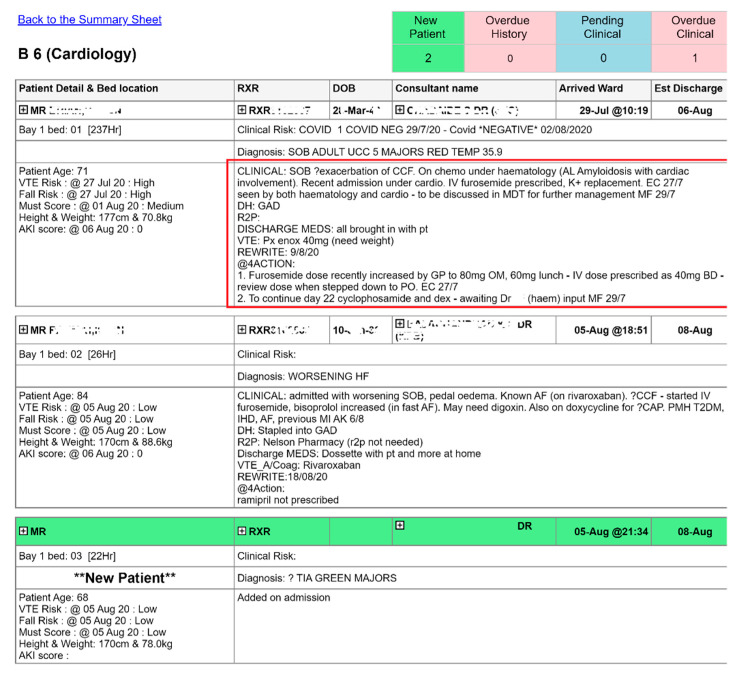
Extract of ward communications sheet (anonymised).

Each patient also had a pharmaceutical care plan added, displayed on a communications sheet for each ward, which pulls patient demography in bed ascending order. This provides pharmacists and technicians with a tool to instantly identify which patients require a pharmacy intervention. Over time, there have been several iterations of this report, with Figure 3 showing the latest version, which now pulls information from three other clinical systems in addition to the EPTS.

**Figure 4:**
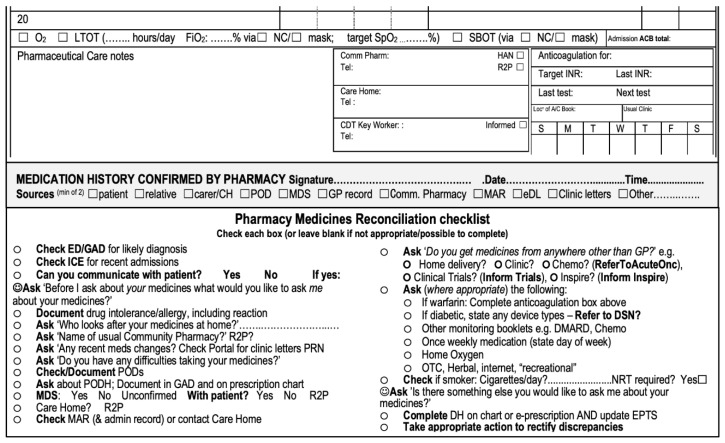
East Lancashire Hospitals NHS Trust medicines reconciliation checklist.

Drug histories are captured in a patient’s notes in a structured manner, utilising a medicines reconciliation checklist. The concept of checklists building quality into a process was extolled by Atul Gawande [8], and we have adapted this approach into many of our processes rather than relying on people remembering to ask all appropriate questions in a rational order. Figure 4 shows the current version, which prompts pharmacists and pharmacy technicians to ask the right questions in a logical order to elicit the best possible medicines history. There were several iterations of this checklist before we arrived at the current one, with early versions being manually inserted into a patient’s notes as we evolved an optimal solution, which is now hard-printed into the generic admissions document, i.e., patients’ notes.

Person-centred care questions specifically supporting medicines reconciliation have been incorporated into our process [9]; these are highlighted in yellow in the image. We intentionally made the approach person centred, as this is a central tenet of NICE’s medicines optimisation guidance and also features heavily in the General Pharmaceutical Council’s Standards for Pharmacy Professionals [4,10].

**Figure 5:**
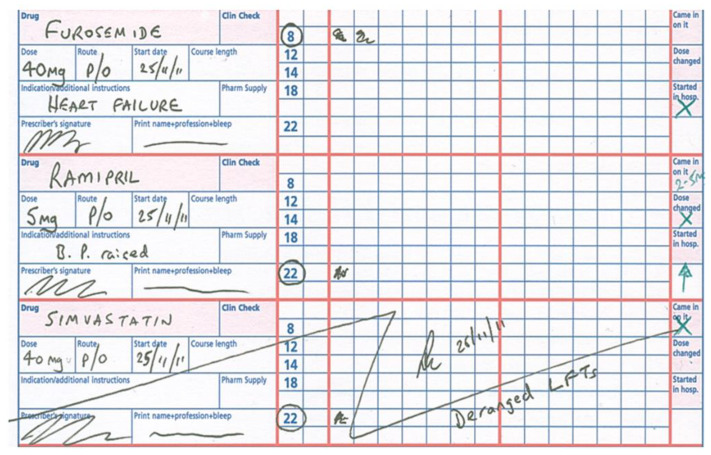
Extract from a transfer of care-‘friendly’ prescription chart.

Our prescription charts have been made transfer of care ‘friendly’, so one can instantly see whether a prescription is new, changed, or whether the patient was admitted on that medicine (Figure 5). A similar approach has been taken regarding the structure and content of our discharge letters.

East Lancashire Hospitals NHS Trust (ELHT) uses Sunquest’s ICE system, which, although not an electronic prescribing system, does allow electronic communication to doctors’ practices and community pharmacies. The letter templates we use for specialties have iteratively changed over time to meet the requirements of the Professional Records Standards Body [11] and also the needs of a specialty, e.g., Obstetrics have a different template to Medicine and Surgery. Discharge summaries are formally referred to as transfer of care letters, although meaningless colloquial terms such as ‘to take out’ (TTO) still persist.

**Figure 6:**
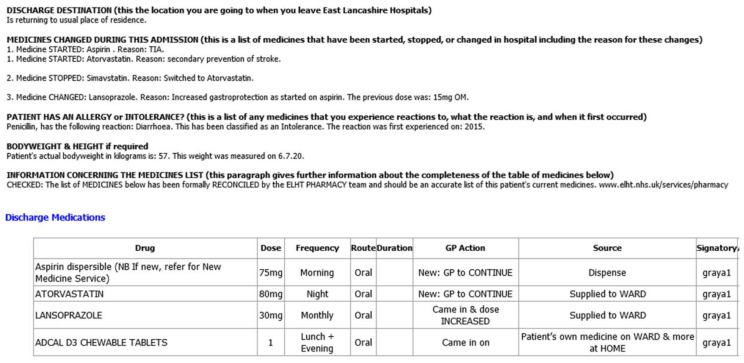
Extract from an East Lancashire Hospitals NHS Trust transfer of care letter (illustrating medicine elements).

With the introduction and spread of dedicated ward pharmacy [12,13], from 2016 to 2019, our wards have had substantial investment to provide the appropriate skill mix of pharmacists, pharmacy technicians and pharmacy assistants. For the majority of wards, this means one pharmacist per ward, and either a dedicated ward pharmacy technician or one that is shared between two adjacent wards. This results in pharmacists participating in over thirty daily consultant-led multidisciplinary ward rounds in order to influence prescribing choices and safety, resolve medicines reconciliation queries, and gain intelligence on which patients are to be discharged in the near future. Liaising with their pharmacy technician, they arrange for the medicines’ elements of transfer of care discharge letters to be created (Figure 6) with prescribers’ signatures obtained if medicine supplies are required. This improves the quality of information captured in the first instance; and reduces the time burden on junior doctors who historically would have created these sections, with a high probability of introducing a prescribing error [14].

## 3. Dedicated Ward Pharmacy Summary Report (1. Improving Communication; 2. Generating Data for Performance Evaluation/Decision Making; 3. Addressing Regulatory Needs; 4. External Patient Driven; 5. Addressing Internal Productivity and Decision Making)

With the evolution of the dedicated ward pharmacy service, there has been an ever-increasing range of dataset points to analyse and act upon. One issue we had was how to provide the senior pharmacists with an overview of the various strands of workload on wards in order to provide assurance that the service was being delivered effectively in order to identify areas where support may be needed acutely, and for ward pharmacists to manage and prioritize their daily workload.

**Figure 7:**
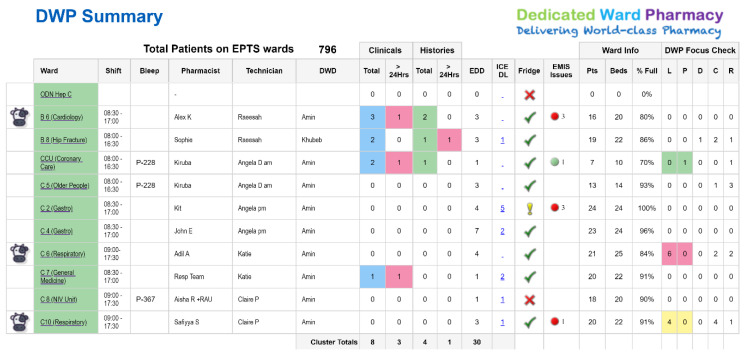
An example of the daily dedicated ward pharmacy summary report for a cluster of wards.

Support was provided to develop these tools from the Trust’s informatics team. Previously, performance data was monitored within a centralized senior management team structure. In order to engage the ward-based pharmacy teams and encourage ownership of performance data streams, we looked at increasing accessibility of our performance data reports which needed to be in real time, clear and concise. As a result, an informatics dashboard (the DWP summary) was developed and made available to all team members via a SharePoint reporting tool (Figure 7).

As this dashboard was iteratively developed, we included further quality assurance measures, such as the daily interactive fridge temperature checks as per Care Quality Commission requirements (the ‘fridge’ column in the figure). In addition, we have included feeds from several systems, e.g., electronic medicine management ordering issues (‘EMIS issues’ in the figure), which wards have static label printers or mobile pharmacy carts (the COW icon), estimated and actual data regarding discharge activity as well as some manually input activity data (EDD, L, P, D and C columns in the figure).

**Figure 8:**
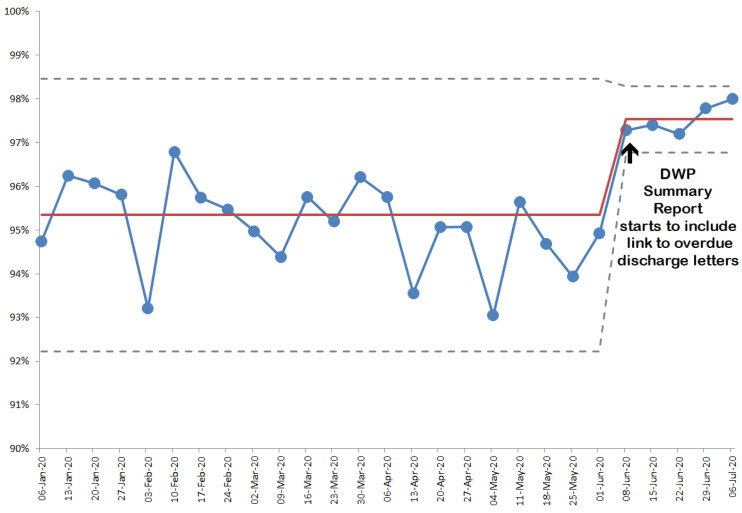
Transfer of care (discharge) letter completion rates showing effect of including this data in the DWP summary report for the ward pharmacists to utilise.

The report has enabled teamworking, to collectively tackle the admission and discharge workload. Another utility is the addition of a report to identify the numbers of patients on each ward who have been discharged without a discharge letter in the last 28 days (the ‘ICE DL’ column in the figure). The pharmacists now utilise this element to drill down to reveal which patients are affected; and ensure that the necessary action is taken so that all patients have a transfer of care letter completed within 1–2 days of discharge (if they have unintentionally been discharged without one). Figure 8 shows the change that has occurred since this link went live; and there is still work to do to achieve 100%.

## 4. Refer-to-Pharmacy (1. Improving Communication; 2. Generating Data for Performance Evaluation/Decision Making; 4. External Patient Driven; 5. Addressing Internal Productivity and Decision Making)

Involvement with a Royal Pharmaceutical Society (RPS) early adopter programme in 2011/12 led to a deep understanding of the problems and solutions to support transfer of care [15]; this directly led to the innovation at ELHT of the world’s first hospital to community pharmacy electronic referral system with increased functionality and integration: Refer-to-Pharmacy. The system went live in 2015 and its genesis also contributed towards an RPS publication to support spread of the concept [16].

We were trying to solve the problem of how to get a discharge transfer of care letter into the hands of patients’ community pharmacists efficiently, reliably and in a timely manner. Early attempts utilizing leaflets and verbally signposting patients had proved ineffective both in the Trust and at other sites taking part in the RPS’s programme. An electronic solution seemed an obvious solution; and it would have to be quick and user friendly in order to gain acceptance at the hospital and community ends. We *just* had to source funding, find a developer, create and test the application, develop an education and communication programme for the pharmacy teams involved, and spread it to all pharmacies in the local health authority, which we did [17].

Refer-to-Pharmacy allows pharmacy team members to make rapid referrals to a patient’s community pharmacy or to the domiciliary Medicines Support Team, either on admission or at discharge. Referrals take a few seconds to complete, making it feasible to refer every eligible patient, with no unnecessary keying or re-keying of information. Recipients receive a notification email to log in to their end of the system where they can manage the referral and view the whole transfer of care discharge letter; they get the same information as a patient’s doctor, at the same time. We have made several screen-grab films of the application which are available on YouTube by searching for Refer-to-Pharmacy.

Eligibility for referral is determined at ‘the point of need’, e.g., on admission, we send hospital admission notifications for Care Home residents and patients who use Multiple Compartmental Aids (MCAs—or ‘blister packs’). Patients who have changes to medicines for long-term conditions are eligible for the New Medicine Service or a Medicines Use Review, and anyone else, where there is a good reason to let the community pharmacist know about a hospital admission or changes to medicines is referred. Referrals for a home visit from the Medicines Support Team are for people who may have difficulty accessing a community pharmacy.

**Figure 9:**
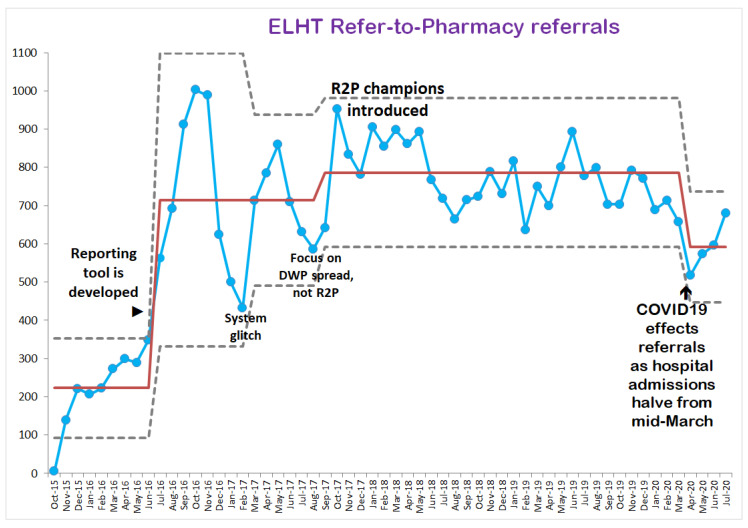
Refer-to-Pharmacy (R2P) referral numbers over time.

Since the system went live in October 2015, there have been 37,628 referrals to the end of July 2020 (Figure 9). Community pharmacists are prompted to record details of any discrepancies that they spot between a transfer of care letter and the first prescription from the patient’s doctor, as well as how many prescriptions have not been dispensed (and medicines wasted). Evaluating recorded outcomes is trickier, as it is reliant on community pharmacists positively recording data when they complete a referral episode, rather than selecting an easier-to-complete neutral response.

There is, however, a very strong signal coming through that each year hundreds of medicines are not being wasted—hundreds of hours are being saved through avoiding dispensing for hospital admitted patients, and annually over one hundred safety incidents are prevented by community pharmacists spotting prescribing anomalies on the first GP prescription after discharge. An example of the latter was a patient discharged following an admission with a gastric bleed caused by taking the oral anticoagulant rivaroxaban. The rivaroxaban was stopped; this was clear on the transfer of care letter sent to both the patient’s doctor and community pharmacist at discharge. The GP unintentionally did not action the change and authorized a repeat prescription for rivaroxaban. The community pharmacist queried this and the GP, realizing the mistake, requested the prescription be cancelled. Before Refer-to-Pharmacy, the community pharmacist would have been unaware of the change and would have dispensed a repeat prescription for rivaroxaban without challenge, which the patient may then have taken with potentially bad consequences.

## 5. Social Media (1. Improving Communication)

During the development phase of Refer-to-Pharmacy, we realized that a communications plan was needed to let people outside the Trust know what was being developed, as it was envisaged that there would be interest from outside of the organisation. The ELHT communications team facilitated the creation of Facebook and Twitter accounts under the @ReferToPharmacy moniker. This was in 2013, and over 2000 people have followed the Twitter feed since then; Refer-to-Pharmacy has acquired a YouTube channel, an Instagram account, and an occasional newsletter (subscribe at: bit.ly/R2PNewsletter).

Those who use social media will appreciate its pros and cons, and an overwhelming pro is the ability to identify and spread good practice and for people to connect who historically would not have done. Within ELHT Pharmacy, there has been a spate of additional Twitter handles created by the various specialist pharmacists, e.g., @ELHTAntimicrob, @ELHTPharmacy, @EDPharmacyELHT, @ELHTMedsSupport, and @ELHTDermPharm. We have utilised the phrase #DedicatedWardPharmacy in many of our posts, and December 2020 saw a daily post on Twitter and Facebook featuring #GalenThePharmacyElf, which doubled as a serious way to illustrate the size and reach of the service, and was also fun to do.

Social media has been used internally via another key enabler with an application called Slack. During induction, our Lead Pharmacy Technician observed numerous failed attempts at the team trying to communicate with one another. The main issue was that the clinical team would often be bleeped by the dispensary, then they would be unable to call back immediately, and then a call back sometime later after the dispensary staff had changed over meant that nobody would know who had bleeped the caller. This all compounded to some notable delays to the completion of discharge or urgent medicines. There was also no way of sharing information with the whole team quickly; when a team is so large and widespread, they will not be able to come together, even for a meeting. As Trusts are managing challenging bed capacity issues daily, it became essential to get announcements out to everyone in real time. This was when we came across “Slack”, which is a backronym for ‘Searchable Log of All Conversation and Knowledge’.

Slack works as a traditional internet chatroom, with the ability to direct message. A web and mobile app are available for staff to work on using laptops on wards and their mobile phones if they wish. In the first 12 months of introducing Slack, over 85,000 messages were sent and received by pharmacy personnel. By uploading rotas to Slack, staff quickly got on board with using it as there go-to form of communication when face to face was not an option. Bleeps are almost obsolete, except at weekends when nursing staff use them to contact the pharmacist or technician who may be covering four or five other wards.

## 6. Dedicated Ward Pharmacy and Intervention Recording (2. Generating Data for Performance Evaluation/Decision Making; 5. Addressing Internal Productivity and Decision Making)

The power of capturing and interpreting the pharmacy team’s interventions has been described elsewhere [12]. This was critical to our understanding of why outcomes such as reduced length of stay, earlier discharges, and reduce readmissions were found during the pilot phase of the service; together, this provided all the evidence we needed for a robust business case.

At the time, we used a tool called Unity to quickly capture and categorize each team member’s inputs. We do not routinely record interventions now unless there is a clear purpose, such as supporting a business case. However, we would now use Microsoft Forms in preference to Unity, as it is even quicker for pharmacy staff to capture their activity, and easier to administer the background settings; both systems export their results into Excel for interpretation and analysis.

## 7. Electronic ‘Forms’ (1. Improving Communication; 5. Addressing Internal Productivity and Decision Making)

Microsoft Forms, as mentioned above, is a really useful survey tool that we are relatively new to. However, since we have come to grips with it, we now use it widely in the department to collate various streams of data that we require on a regular basis, e.g., antimicrobial audit. We also use it to do quick staff surveys such as who has undergone ‘fit mask testing’, or what size uniform do you need (pharmacists were allocated tunics and trousers during the pandemic, and we had 80 returns within 24 h).

During the COVID19 pandemic, we needed a method of communicating between our aseptic manufacturing unit and the members of the pharmacy team who were donning full PPE in the critical care areas, which had more than doubled in size. The use of Google Forms, and latterly Microsoft Forms (we switched to this with the roll out of Office 365 across the Trust), allowed the seamless, paperless and timely transfer of key information on the number of aseptic products required for the next 24 h period. This allowed the aseptic unit to prioritize their workload and reduced the amount of waste by preventing products being unnecessarily manufactured and expiring.

**Figure 10:**
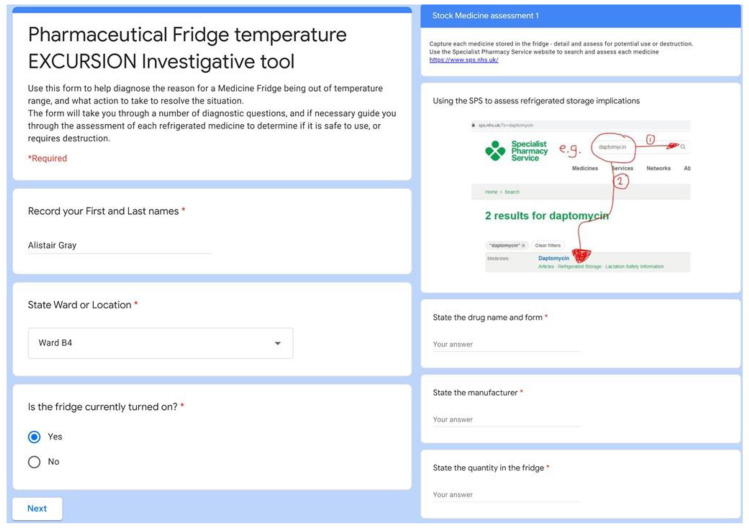
Screenshots from the East Lancashire Hospitals NHS Trust transfer pharmacy fridge temperature excursion tool.

Another great use of this approach was in diagnosing and collecting information if a clinical area has a fridge temperature excursion (Figure 10). The form replaced a paper version and is a valuable tool, not only to collect the data to provide assurance that fridge temperature excursions are managed appropriately, but also to direct certain actions to be undertaken depending on the responses inputted by the user. The form acts as an interactive algorithm so nothing is missed. To support staff with day to day temperature monitoring, we made some short films showing how to read and reset maximum and minimum temperatures on the various fridges in the Trust (viewable on the Refer-to-Pharmacy YouTube channel).

## 8. Electronic Medicines Management (1. Improving Communication; 2. Generating Data for Performance Evaluation/Decision Making; 5. Addressing Internal Productivity and Decision Making)

There has been a long-standing relationship between ELHT and EMIS Health (and prior to that, Ascribe), with the Trust acquiring several years ago Electronic Medicines Management (EMM), which is a module that works alongside the pharmacy dispensing program although its main utility is to support ePMA. At the time, we were about to go live with an ePMA system and the module was going to be critical to integration between the two systems. 

Unfortunately the WannaCry cyberattack in 2017 led to our potential ePMA system being halted. However, in the absence of electronic prescribing, it still has a utility in allowing ward-based pharmacy staff to add supply requests for medicines to work lists, which are visible to the dispensary team. Dispensing then occurs by entering patients’ medication records from the notifications in these work lists. This allows for a paperless transmission of supply requests to the dispensary from the wards, which solved an issue we did have regarding the legibility of hand-transcribed medicine supply requests and the timeliness of getting those paper requests down to the dispensary and dispensed, or sending prescription charts down to the dispensary (that was particularly prevalent at weekends), which meant that they were not available on wards when they needed.

**Figure 11:**
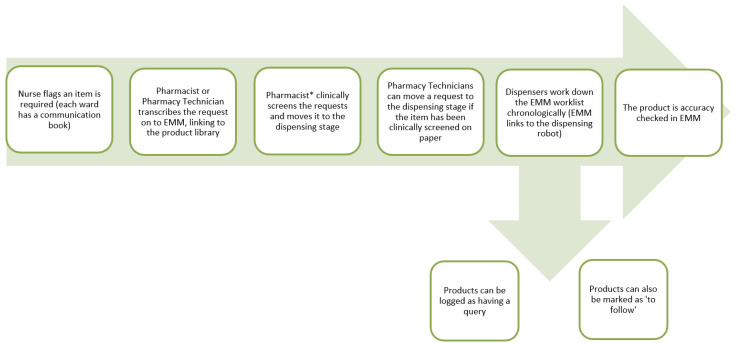
Medicines supply workflow using electronic medicines management (EMM).

The benefit of this method means prescription charts stay on the ward, information transfer is immediate, and there is no delay in getting the order to the dispensary. EMM is configurable to an extent to meet the needs of our processes, with the current workflow shown in Figure 11.

This paperless way of managing work created an unsighted workload for the dispensary team; we could no longer make a quick assessment of what dispensing work was left, and what work was expected, and we no longer had trays of work and piles of medicines coming through the dispensary. Whilst this was refreshing, it also left staff feeling uneasy; there is comfort in seeing the mountain left to climb, so we set about acquiring a tracking system.

**Figure 12:**
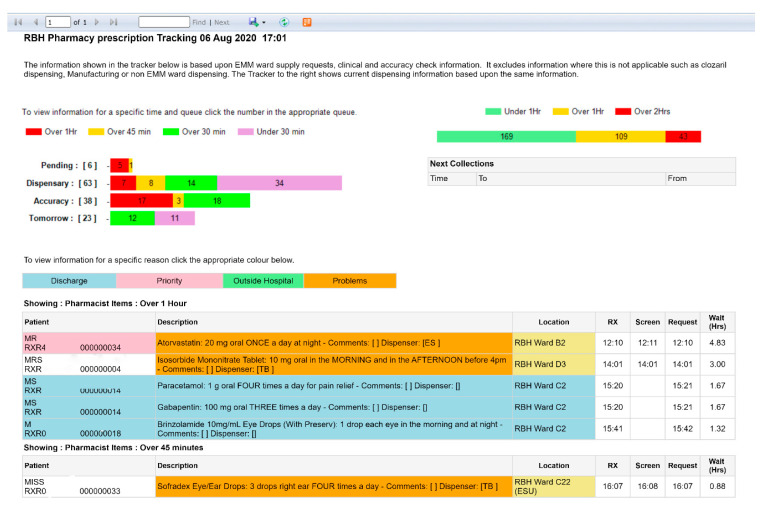
Prescription tracker at East Lancashire Hospitals NHS Trust.

Commercial options were available, at costs which we could not justify. However, we were able to build a solution on an intranet page which pulled the worklist data from EMM (Figure 12). The data is currently only visible to pharmacy staff, displayed on a large monitor in both dispensaries to help staff have an oversight of what work requires a clinical check, how many items need to be dispensed, and how many items need accuracy checking. This data helps make quick decisions around staffing, freeing up ward-based staff to remain on wards if they are not needed, and it also allows staff to return to the dispensary to support peaks in workload. The data is also the source of one of the feeds on the DWP summary report.

The dedicated ward pharmacy model means that pharmacists gain good intelligence of who is going home on any given day, and who is expected to leave in the coming days. This means that pharmacy technicians and assistants can assess likely dispensing needs in advance and make supply requests on the ‘Tomorrow List’ in EMM. This allows more flexibility in dispensing, meaning more urgent items can be supplied first. 

**Figure 13:**
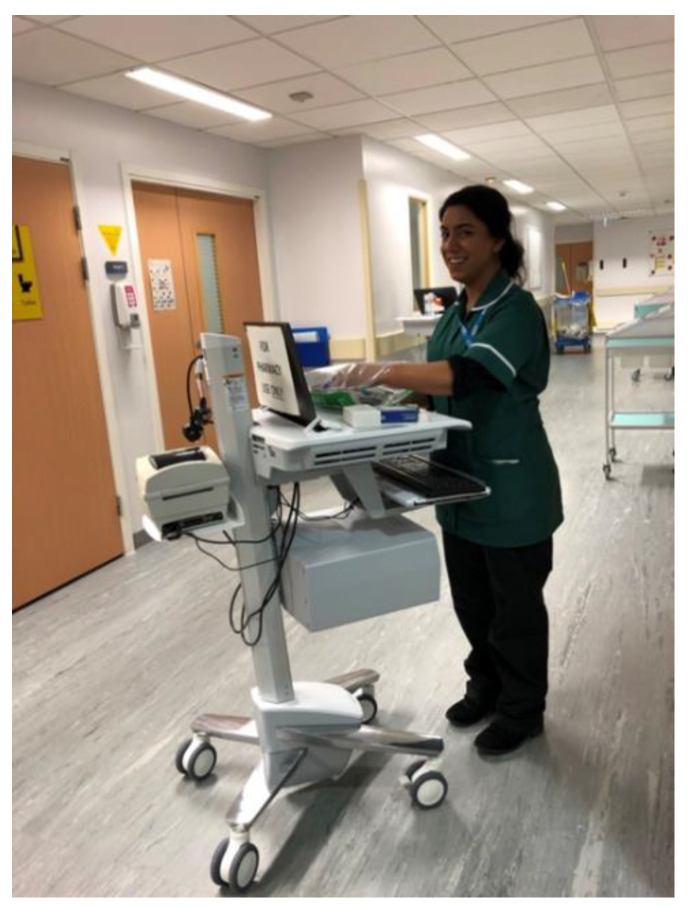
East Lancashire Hospitals NHS Trust pharmacy technician with one of the pharmacy carts.

A number of years ago, we also strategically spread ten labelling machines around the Trust, so there is always one near a ward someone is working on to facilitate re-labelling, or near to patient dispensing using ward stocks or sumps of additional stocks for predictable activity on wards such as cardiology. Recently, we have moved six of the static labellers on to Ergotron^®^ carts to give team members more flexibility with their working surfaces (Figure 13).

## 9. EMM Shortcuts (1. Improving Communication; 5. Addressing Internal Productivity and Decision Making)

Having over 120 pharmacists and pharmacy technicians all using and sharing laptops, tablets and PCs presents a problem, especially when they need to open several applications or webpages to have information quickly at their fingertips. Yes, the Trust has a ‘Favourites’ menu but that is too broad for the pharmacy team’s needs. It turned out that we could embed common links in the pharmacy system, so now all commonly used resources, programs and applications, e.g., the Trust’s antimicrobial app, renal drug database, or Toxbase can be launched from one page that all the pharmacy team can access. 

**Figure 14:**
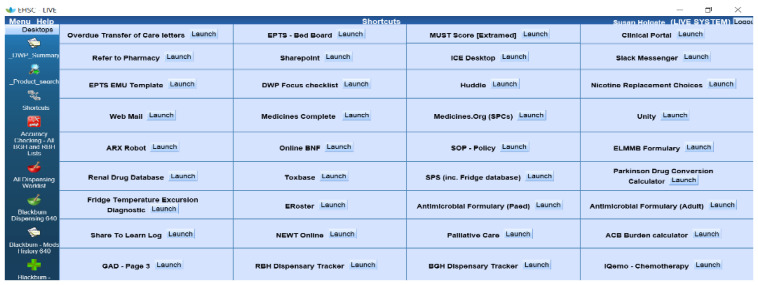
Screenshot of the shortcuts menu on the pharmacy system.

It also houses links to Word documents that we commonly use in our day to day practice, e.g., the EPTS template we use to input our pharmaceutical care plans. It is configured to our needs; we can add to it, change it, and update it as our practice changes. Initially, it started out as a couple of links and now houses over twenty (Figure 14).

## 10. Clinical Portal (1. Improving Communication; 3. Addressing Regulatory Needs; 5. Addressing Internal Productivity and Decision Making)

Most organisations will use the summary care record (SCR), but in ELHT we have been utilizing our Clinical Portal, which draws data down from the Lancashire Person Record Exchange Service (LPRES). This health application covers the health economy of the Lancashire and South Cumbria Integrated Care System (LSC ICS) and pulls through patient data from over 300 GP practices as well as outpatient and hospital discharge letters, providing a richer vein of information than SCR currently does.

We are contributing to the next iteration of LPRES at the time of writing. The aim is that “LPRES 2.0” will pull medicines data from all other ICS sources of prescribing, e.g., clozapine clinics and community drugs teams, and possibly community pharmacy dispensing records in order to create a unified medicines record available at the point of need. That point of need depends on whether a patient is referred to a service or presents at one, and we want to ensure that both scenarios are optimized to produce the best health outcomes.

Part of this vision is to provide an ED (or admissions wards’) viewer, so that all patients on those units can have a high-level screen to identify who is usually prescribed ‘critical medicines’, and therefore most in need of a pharmacy intervention to avoid a dose omission. This addresses the issue of seeing patients in chronological order, or by chance; we will be able to target patients who are potentially the most vulnerable.

## 11. Critical Care Injectables and Fluid ‘App’ (5. Addressing Internal Productivity and Decision Making)

The idea of creating an ‘app’ to house critical care-specific drug monographs and guidelines came about pre-pandemic. However, the Trust-wide opportunities for what we developed became apparent during COVID-19, when it was crucial to collaborate and standardize guidelines in each specialty. This is particularly an issue to medical and nursing staff who have to make up injections and infusions and do not always have easy access to the information they need. Trust monographs held on the intranet are not accessible in ward treatment rooms, whereas many staff do have mobile phones on their persons.

**Figure 15:**
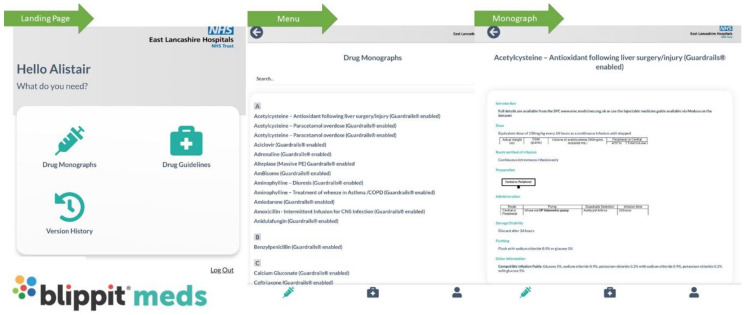
Screenshots from Blippit^®^ meds.

Our ‘app’, Blippit^®^ meds (Figure 15), currently holds a library of drug monographs used by critical care, outlining in simple terms: the recommended dose of a drug, how to reconstitute and administer it, what rate to administer and any incompatibilities that are prudent to safe practice. The ‘app’ enables dosing tables and illustrations to make the content user friendly and simple to follow, and also holds a whole host of clinical guidelines, which enables prescribers, allied health professionals, and nursing staff access to information and advice on safe and effective medicines management.

Blippit^®^ meds is now being expanded to accommodate a whole range of specialties, including emergency medicine, respiratory, cardiovascular, gastroenterology, endocrinology, surgery, paediatrics and more. The ‘app’ will house clinical decision-making tools, supported by national guidelines, particularly for Fluid Stewardship and insulin dosing. It is hoped the ‘app’ will be shared nationally as an example of good practice and excellent use of technology to deliver healthcare.

## 12. Emergency Department Pharmacy Service (1. Improving Communication; 2. Generating Data for Performance Evaluation/Decision Making; 4. External Patient Driven; 5. Addressing Internal Productivity and Decision Making)

The aim of our Emergency Department (ED) Pharmacist is to screen all patients admitted into the ED Majors and Resuscitation areas during her shift to identify what regular medicines they were taking at home in order to prioritize which patients needed the most urgent pharmaceutical attention, especially those taking ‘critical medicines’ (Parkinson’s Disease, insulins, anti-epileptics, etc.). This was very difficult to achieve given the high turnover of patients and fluidity of patient movement within the department. The ED does, however, utilize the EPTS, albeit an ED module which has no pharmacy-specific functionality; it is utilized mainly by other ED staff to update Early Warning Scores and create bed requests for admissions, or to discharge the patient.

**Figure 16:**
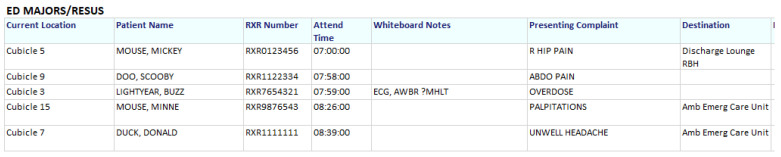
Example of the ED pharmacy communications sheet.

This issue was raised with the informatics team with the challenge of whether they could create a printable ED pharmacy communications sheet which pulled live information from EPTS about the patient’s location, hospital number, name, time of arrival to the department, presenting complaint and discharge/ward destination. Figure 16 shows an example; having this information has saved a lot of time that previously was written down by hand.

As part of an ED pharmacy service pilot, the ED Pharmacist completed a night shift. She noticed that doctors would write down all the patients who were still in the department that required handing over to the day team onto a piece of paper, along with their presenting complaint and their ‘SBAR’ information [18]. This could take anything up to two hours, in which the time could be better utilised to review more patients and prevent breaches within the department.

This led to another conversation with the informatics department, this time asking whether they could create another ED communications sheet with information tailored to the doctors’ requirements at handover. Once this was created, it turned out that not only do the doctors find this beneficial, but the ED nurses have found an even greater use for it!

## 13. Electronic Drug Monitoring Software (5. Addressing Internal Productivity and Decision Making)

Secondary care specialist services such as Dermatology, Gastroenterology and Rheumatology provide patients with a range of options for the management of complex conditions. These include medicines that require close monitoring to ensure that they are used safely and are effective. The importance of expertise from specialist teams in managing such regimens is recognized in national and international guidance [19,20,21]. In practice, however, there is an issue around the manual monitoring of laboratory results for patients on high-risk drugs that can require the devotion of a significant amount of the clinical team’s time. Carrying out the check for each patient requires looking them up on the laboratory system and, in the majority of instances, the results are within acceptable bounds and no further action is needed.

**Figure 17:**
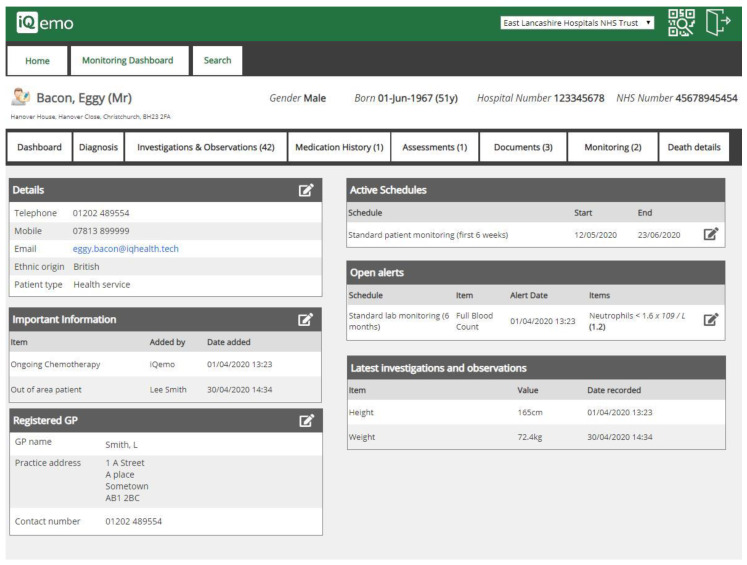

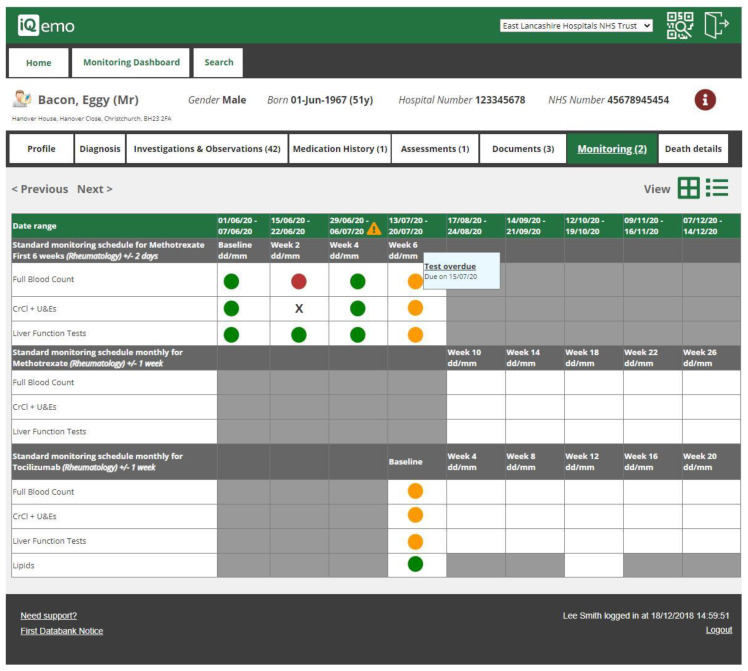
Example of IQ HealthTech’s drug monitoring software at East Lancashire Hospitals NHS Trust.

ELHT is working with IQ HealthTech to develop drug monitoring software that will automate the checking process. Monitoring schedules for each high-risk drug are programmed into the system and patients allocated to the appropriate monitoring schedule. The software will check all applicable results on a daily basis and only alert clinicians to review patients who have abnormal results, or patients who have not obtained an expected result on time. The software will also use the Trust communication capabilities to send out SMS text reminders to patients who are due to have a test or who have missed a deadline for an expected test. This automation should result in fewer patients missing out on monitoring and reduce the amount of time clinicians spend on menial activities to check results. This system will also have a variety of side benefits such as providing easy access to associated demographic data for the patients and providing a fully auditable trail of responses to the various abnormalities that can occur. Figure 17 shows a copy of a test patient.

## 14. Homecare Prescription Tracking Tool (1. Improving Communication; 4. External Patient Driven; 5. Addressing Internal Productivity and Decision Making)

The ELHT Pharmacy Homecare team provides a link between the Trust’s prescribers and third-party homecare companies to facilitate the supply of drugs to patients’ homes that are often high cost and require careful stock management. Acting as an interface between the Trust and a variety of outside companies can lead to confusion—in particular, there are issues over deadlines for supply and keeping track of what paperwork is needed when, and by whom.

**Figure 18:**
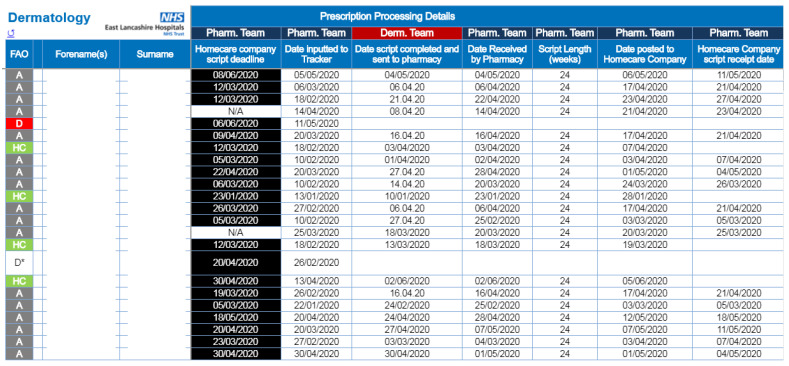
East Lancashire Hospitals NHS Trust’s homecare prescription tracking tool.

To resolve the situation, the ELHT Pharmacy Homecare team now make use of the Trust’s new cloud-based storage to manage a shared database of patients and prescriptions. This ‘Prescription Tracking Tool’ uses Trust login credentials to limit access only to staff members involved in the management of prescribing and supply (Figure 18). All approved users can simultaneously access the tracking tool and see live updates on the progress of prescriptions. The tool highlights which staff group needs to take the next action to progress a prescription and allows the user to see filtered results of only the patients where their input is required.

By keeping all of the data in one place, it not only makes it easier to see what action needs taking for each patient at a glance, but also allows for extracting data to produce statistics on a regular basis, something that had hitherto been carried out with a hand count of paper prescriptions by the Pharmacy Homecare team.

## 15. Impact on Wider Practice and The Future

Our work has been brought to wider attention through various journal articles and conference speaking opportunities. This has led to many other Trusts and organisations getting in touch or visiting in order to discuss how we did what we did and in particular to see dedicated ward pharmacy in action. To date, a handful of Trusts have been inspired to institute their version of this service. This is heartening, as too often innovation in healthcare is rife but spread of good practice is rare. Spreading good practice is something that we have been keen to address since our collaboration in the RPS transfer of care early adopter programme nearly a decade ago [15].

We hope this gives a soupçon of some of the ways we have utilized the technology available to the East Lancashire Hospitals pharmacy team, and how we have integrated it with the all-important human interface. We do not stop; we will continue to build on the systems we have, finding better, more efficient ways of doing things. This has been our modus operandi to date, combined with quality improvement science approaches. Hopefully an ePMA and ePR will arrive in the not too distant future. In the meantime, get in touch if you would like to find out more, or keep in touch by subscribing to the ‘R2P newsletter’.
